# Predictive modelling to differentiate possible bacterial and viral cases of childhood pneumonia in Kilifi, Kenya using protein markers and clinical data

**DOI:** 10.3389/fmed.2026.1943481

**Published:** 2026-10-20

**Authors:** Cynthia Matuli, Jacqueline M. Waeni, Elijah T. Gicheru, Charles J. Sande, Katherine Gallagher

**Affiliations:** 1 Epidemiology and Demography Department, KEMRI-Wellcome Trust Research Programme, Kilifi, Kenya; 2 Bioscience Department, KEMRI-Wellcome Trust Research Programme, Kilifi, Kenya; 3 Nuffield Department of Medicine, University of Oxford, Oxford, United Kingdom; 4 Department of Infectious Disease Epidemiology, Faculty of Epidemiology and Population Health, London School of Hygiene and Tropical Medicine, London, United Kingdom

**Keywords:** bacterial pneumonia, viral pneumonia, biomarkers, diagnostics, performance metrics, pneumonia differentiation, pneumonia etiology

## Abstract

**Background:**

To date, accessible diagnostic tools to identify whether a patient's pneumonia is a bacterial, or viral infection, are not accurate or timely enough to prevent preemptive antibiotic administration. Relying on single biomarkers or clinical presentations has been insufficient. We aimed to incorporate a wide range of novel biomarkers and clinical presentations into a multivariable model and to validate its ability to differentiate bacterial from viral pneumonia.

**Methods:**

A total of 457 children aged 2–59 months, admitted to the Kilifi County Referral Hospital, Kenya, with WHO defined pneumonia were classified using gold standard laboratory diagnostics as possible bacterial (i.e., bacterial/ unknown cases, *n* = 229) or possible viral (*n* = 228) infections. Clinical and laboratory data, and levels of CRP, PCT and 6 novel protein biomarkers were used to develop and validate a predictive multivariable Poisson regression model to differentiate pneumonia etiology. The Receiver Operating Characteristic curve was used to assess biomarker performance and validate the model internally.

**Results:**

In age-adjusted analyses, chest-wall indrawing, cough, convulsions, crackles, angiotensinogen (AGT), and Serpin Family A Member 1 (SERPINA1) were significantly associated with pneumonia etiology, controlling for age. However, only chest-wall indrawing remained significant in multivariable analyses after controlling for age. The model demonstrated fair, but inadequate, discrimination, with an Area Under the Curve of 0.61. In a separate analysis excluding the unknowns from the bacterial group, chest-wall indrawing, crackles, cough, AGT (>=7.9144MFI), were significant predictors of pneumonia etiology, adjusting for age. This model demonstrated good discrimination, with an Area Under the Curve of 0.78 on the validation dataset.

**Conclusion:**

Among children admitted to the hospital with WHO-defined pneumonia, modelling a wide range of biomarkers and clinical presentations still failed to distinguish bacterial from viral pneumonia with enough accuracy to guide clinical management. Further research addressing some of the limitations we encountered in outcome classification, stratifying by disease severity and potentially accounting for variation in biomarker performance by age, may improve predictions.

## Introduction

Globally, the incidence of pneumonia in the pediatric population has significantly decreased following the widespread administration of pneumococcal conjugate vaccines (PCVs) and *Haemophilus influenzae* type b (Hib) vaccines (1, 2). However, pneumonia remains one of the leading causes of pediatric morbidity and mortality, particularly in sub-Saharan Africa. As bacterial infections have decreased, severe viral infections now account for a larger proportion of the hospital caseload (1, 3). Guidelines still recommend the empirical use of antibiotics, as this has been instrumental in preventing bacterial infection-related deaths; however, it is thought that unnecessary use of antibiotics for viral infections has inadvertently fueled antimicrobial resistance (AMR) (4, 5). Diagnostic tools to guide the rational use of antibiotics in the treatment of pneumonia are needed.

The distinction between bacterial and viral pneumonia remains challenging, especially in Low- and Middle-Income countries (LMICs). An accurate distinction between bacterial and viral pathogens is crucial for guiding antibiotic treatment (5). However, conventional methods to identify bacterial infection, such as blood and sputum cultures, are invasive, have limited sensitivity, and are time-consuming (6, 7). Meanwhile, molecular testing, such as Polymerase Chain Reaction (PCR) (6, 7), provides higher sensitivities and specificities, but is expensive and only detects specific pathogens. Transcriptomics has been used to accurately distinguish bacterial from viral pneumonia with high sensitivity and specificity (8, 9); however, it remains impractical in LMICs due to its high cost and the need for advanced laboratory infrastructure (9).

Scalable and more affordable methods to distinguish bacterial pneumonia from viral pneumonia remain elusive (10, 11). Individuals with bacterial pneumonia and those with viral pneumonia present with overlapping clinical signs and symptoms and biomarker levels making the use of individual symptoms or concentrations of solitary markers suboptimal (12–14). Concentrations of biomarkers such as C-reactive protein (CRP) and procalcitonin (PCT), have in the past been significantly higher in bacterial pneumonia compared to viral pneumonia cases (15). However, these biomarkers had low sensitivities (CRP = 0.70, PCT = 0.66). CRP lacks etiological specificity and is elevated by co-infections such as malaria (6, 10, 12), obesity, and cardiovascular diseases (16). PCT can be falsely low in localized infections, such as empyema, due to the time lag between infection onset and its peak, and is also elevated by coinfections, surgery, or trauma (11, 17). White blood cell counts (WBC), absolute neutrophil count (ANC), and monocyte values vary by ethnicity, with individuals of African ancestry having significantly lower counts, which limits generalizability (18).

Combining a wide range of biomarkers with clinical presentations may result in better diagnostic accuracy (5, 19, 20). Very few studies have attempted this, especially in sub-Saharan Africa and in the pediatric population and the resultant models have had limited predictive capacity, have included only a few serological biomarkers, and have had small study sample sizes (21). This study integrates a wide range of novel protein biomarkers, demographic, anthropometric, and clinical data into a single model to predict whether a child presenting with pneumonia is likely to have a bacterial or viral infection.

## Materials and methods

### Study setting and population

Data on children presenting to Kilifi County Referral Hospital (KCRH) with pneumonia were derived from the long-term surveillance of Respiratory Syncytial Virus (RSV) in childhood lower respiratory tract infection study, conducted between January 2007 and August 2020. This project sampled children less than 5 years of age who were either febrile or presented with sepsis or pneumonia for biomarker discovery and validation (8). Children were excluded if born prematurely, were less than 1 week of age, or had a history of congenital heart disease. For the analysis presented here, this nested case-control study selected data from children aged 2–59 months who met the 2013 WHO definition of pneumonia (22). This study utilized secondary data, and therefore, the patients were participants in the study and were not involved in the design, conduct, or dissemination plan of this study's findings.

### Study procedures

Under the surveillance protocol, anthropometric (e.g., weight, height), clinical (e.g., cough, cyanosis, hypoxemia), and demographic data (e.g., age, gender), blood samples, and nasopharyngeal/oropharyngeal (NP/OP) swabs were collected at admission. NP/OP swabs were used to test for 15 common childhood viral infections, including adenovirus, parainfluenza virus (1,2,3, and 4), RSV (A and B), influenza (A, B, and C), rhinovirus, human metapneumovirus, and coronaviruses (OC43, NL63, and E229) using multiplex real-time PCR. Blood specimens were cultured for bacteria, and a blood slide was prepared to determine malaria infection status. Blood samples were analyzed with a complete blood count [WBC, red blood cell counts (RBC), platelets, neutrophils, eosinophils, and basophils]. The biomarker discovery study tested the blood samples from a subset of children and quantified 418 plasma proteins using high-performance liquid chromatography tandem mass spectrometry (HPLC-MS/MS) [11]. CRP and 6 other plasma proteins appeared predictive of infectious aetiology: angiotensinogen (AGT), Serpin Family A Member 1 (SERPINA1), Serpin Family A Member 3 (SERPINA3), Paraoxonase-1 (PON1), Histidine Rich Glycoprotein (HRG/B2R8I2), and lipopolysaccharide-binding protein (LBP) [11] and were therefore considered for etiology differentiation for this study. Samples from the whole cohort of children were then tested for CRP, PCT, and these 6 novel proteins using the same mass spectrometry. The ILab Aries clinical biochemistry analyzer was used to measure a subset of 100 CRP samples, which were converted to concentrations (8). Laboratory sample testing was conducted at the KEMRI-Wellcome Trust Research Programme (KWTRP) in Kilifi, Kenya.

### Statistical analyses

For the secondary data analysis presented in this paper, pneumonia cases were classified as viral, bacterial, bacterial-viral co-infections, and unknown infections (Figure 1). For clinical relevance, we combined children with evidence of bacterial infections, bacterial-viral co-infections, and unknown infections into a single group (possible bacterial infections) that would need to receive empirical antibiotic therapy if presenting to a healthcare facility. Children whose NP/OP swabs were PCR + for viral infections known to cause pneumonia [adenovirus, parainfluenza virus (1,2,3, and 4), RSV (A and B), influenza (A, B, and C), and human metapneumovirus] were defined as the probable viral pneumonia group.

**Figure 1:**
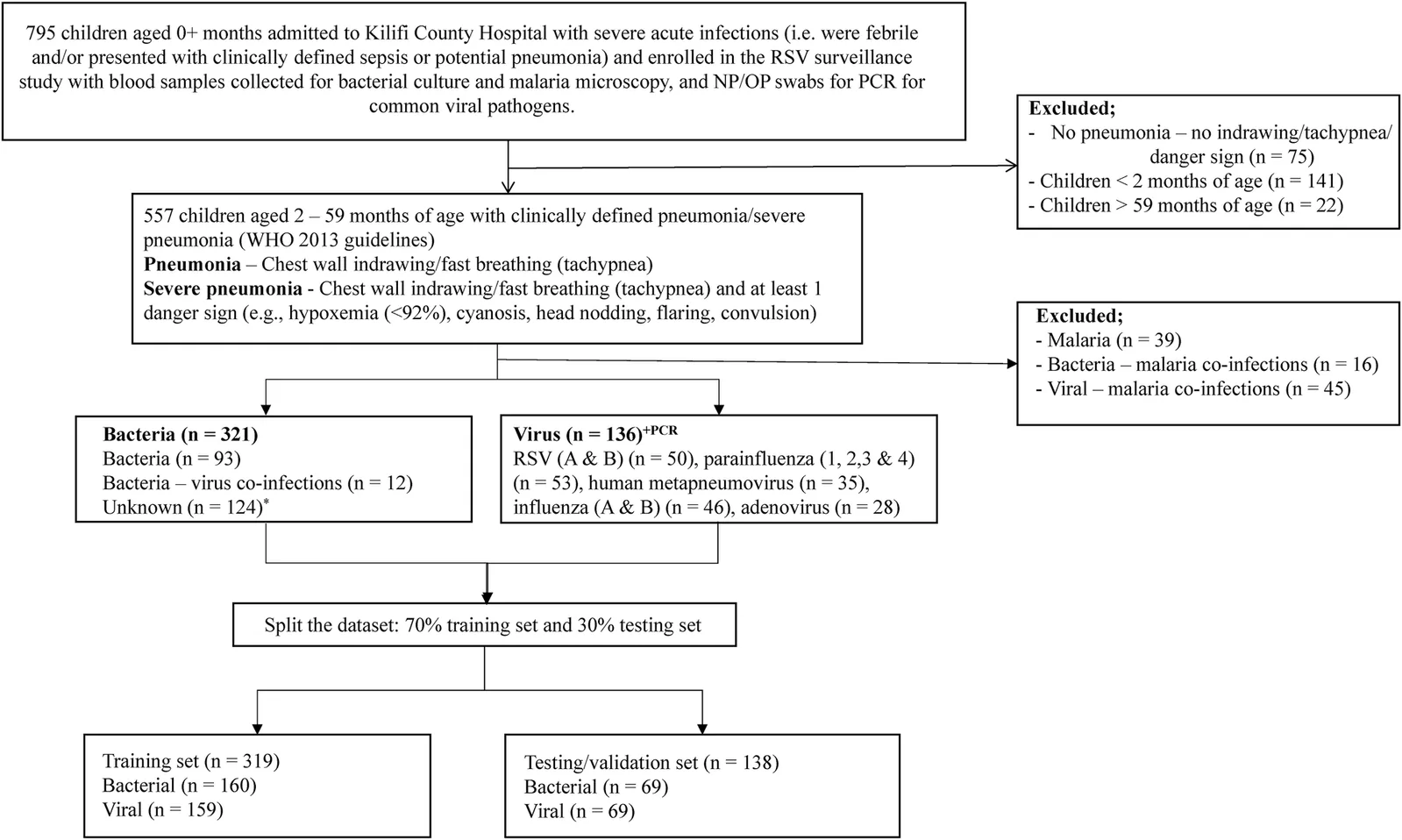
This is the participants’ flowchart. It provides a detailed explanation of the flow by which the study determined the working sample size. It also shows which criteria of participants were eliminated and the pathogens that guided our case definition.

Variables such as cough duration were missing in >25% of participants and excluded from the analysis. Using the training dataset, excluding children with infections of unknown aetiology and bacterial-viral co-infections, the Receiver Operating Characteristic (ROC) curve was used to identify optimal cutoff points for the mean fluorescent intensity (MFI) values of biomarkers, enabling categorization into categorical variables with the highest sensitivity, specificity, and overall discriminatory ability. The cutpointr package in R was utilized to determine the optimal threshold of each biomarker using the Youden index (sensitivity + specificity – 1). To enhance the robustness of the estimates, a 5-fold cross-validation stratified by etiology was used to assess the discriminatory power of each candidate biomarker and to identify the optimal threshold (cut-offs). These derived cut-offs from the training set were then applied unchanged to the validation dataset. Further exploratory analyses to determine cut-off points for biomarkers were also identified by stratifying the dataset by age (<1 and ≥1 years, <2 and ≥2 years, and <3 and ≥3 years) on the entire dataset to assess whether cut-offs varied by age, as this has been reported in some previous literature (26). The Variance Inflation Factor (VIF) was used to evaluate multicollinearity among variables. Highly correlated variables (VIF >2) were assessed for their discriminatory power. The variable with the lower AUC was dropped. Exploratory interaction analyses were conducted after the primary analyses to assess whether an interaction between pneumonia severity and the eight biomarkers, adjusting for age, could explain the biomarkers’ performance. Interaction terms were included in separate modified Poisson regression models and significance assessed using the Wald test with robust standard errors.

### Model development

The dataset was randomly split into two datasets, stratified by etiology; 70% of the data was used for training and 30% for model validation. Age was considered *a priori* as a confounder in every stage of the analysis. A modified Poisson regression model was used to assess the association between every predictor and the outcome. Covariates with a likelihood ratio test (LRT) *p*-value < 0.2 in the age-adjusted analyses were considered to improve model fit and were therefore included in the selection for the multivariable predictive model. The multivariable Poisson regression model was fitted using backward selection. Variables were iteratively removed from the multivariate model, and variables that significantly improved the model's fit (LRT *p*-value < 0.05) were retained in the final model. The overall sensitivity, specificity, and discriminative power (AUC) of the final model for distinguishing bacterial from viral pneumonia cases were assessed. To assess the stability of the model development process and performance, a 5-fold cross-validation, stratified by etiology, on the training set was performed. Within each fold, 4 folds were used as the model development sets, while one fold was held out for out-of-sample model validation. The complete model-building procedure was performed in each fold, adjusting for age. First, each predictor was assessed for its association with etiology, using an LRT *p*-value <0.2 to identify potential predictors for inclusion in the multivariable model. Backward elimination was then performed, and variables with an LRT *p*-value <0.05 were retained in the multivariable models. The models’ performance was then assessed on the out-of-sample validation dataset using the AUC. The overall model performance was recorded as the mean AUC of the 5 cross-validation folds.

### Model validation

The model's ability to distinguish between bacterial and viral pneumonia cases was assessed using Area Under the Curve (AUC) values on the validation dataset. Other published predictive models were also run on the validation dataset to determine comparative discriminatory power. An AUC of 0.5 indicated that the model cannot discriminate beyond chance; a value greater than 0.7 was considered moderate discrimination, while a value greater than 0.80 was regarded as good discrimination (23). The model's calibration was assessed using the calibration intercept, which measures how close the predicted risks are to the observed event (underestimation or overestimation), and the calibration slope, which indicates potential model overfit or underfit (23). We externally validated a previously published model (15), developed in a study of Indonesian children aged 2–59 months with pneumonia (WHO 2014 definition) who were hospitalised within 24 h of admission, by applying their logistic regression model to our dataset without modification. We defined the variables in their final model as in their publication, without refitting or recalibrating it. Another study (9) conducted in Western Australian children ≤17 years of age recommended a CRP cutoff of 72 mg/L in the presence of fever (≥38 °C) or absence of rhinorrhea. We validated these models using CRP data analysed on the ILab Aries clinical biochemistry analyser. All statistical analyses were conducted using R software version 4.5.1, and variable significance was evaluated at a 5% significance level.

### Ethics approval

The long-term surveillance of RSV in childhood lower respiratory tract infections study (SSC 1055) was approved by the Kenya Medical Research Institute Scientific Ethics Research Unit (KEMRI–SERU) and conducted in accordance with the principles of Good Clinical Practice and Good Clinical Laboratory Practice (GCLP). Written informed consent was obtained from the participant's respective legal guardian or next of kin, and the study adhered to the principles outlined in the Declaration of Helsinki. For this nested study, data were shared with the approval of the study Principal Investigator and the KWTRP data governance committee.

## Results

Among the 457 children enrolled in this study, 228 (49.9%) had NP/OP swabs PCR + for viral infections known to cause pneumonia and no evidence of other infections, 93 (20.4%) had a positive blood culture for a bacterial infection, 12 (2.6%) had a bacterial-viral co-infection, and 124 (27.1%) had unknown etiology (i.e., negative blood culture, negative PCR panel, negative malaria slide; Figure 1). Among the 228 with a viral infection, the most identified viral pathogens were RSV-A (*n* = 39, 17.1%), parainfluenza-3 (*n* = 36, 15.8%), human metapneumovirus (*n* = 35, 15.4%), influenza-A (*n* = 34, 14.9%), and adenovirus (*n* = 28, 12.3%). The most identified bacterial pathogens from blood cultures were *Escherichia coli* (*n* = 17, 7.4%)*, Streptococcus pneumoniae* (*n* = 17, 7.4%), *Staphylococcus aureus* (*n* = 11, 4.8%), and *Haemophilus influenzae* (*n* = 8, 3.5%). (Supplementary Figures S1, S2).

### Demographic, laboratory, and clinical characteristics

More than half (63%) of the study population had severe pneumonia compared to (non-severe) pneumonia (37%) (Supplementary Table S2). A higher proportion of children with probable viral pneumonia presented with severe pneumonia (72%), compared to children with possible bacterial pneumonia (54%) (Supplementary Table S2). Cell counts of WBC (*p*-value = 0.002), neutrophils (*p*-value = 0.017), and the median fluorescent intensity (MFI) units of AGT (*p*-value = 0.002), LBP (*p*-value = 0.005), PCT (*p*-value = 0.020), and SERPINA1 (*p*-value = 0.002) were significantly higher in the bacterial group, while the cell counts of RBC (*p*-value = 0.014), hemoglobin (*p*-value = 0.029) concentrations, and MFI units of SERPINA3 (*p*-value = 0.014) were significantly higher in the viral group (Supplementary Table S1).

### Predictive capacity of each biomarker to differentiate bacterial from viral infections

Based on this nested study's case definition, AGT had the best discriminatory power (AUC = 0.60, 95% CI [0.53, 0.66]), followed by PCT (AUC = 0.58, 95% CI [0.52, 0.64]) and SERPINA1 (AUC = 0.58, 95% CI [0.52, 0.64]), and lastly LBP (AUC = 0.56, 95% CI [0.50, 0.63] (Table 1)). CRP, HRG, PON1, and SERPINA3 could not distinguish bacterial from viral infections beyond chance as their confidence intervals had limits below 0.50 (Table S1). Assessing the biomarker performance on ‘pure’ bacterial and viral infections of pneumonia, AGT (AUC = 0.76, 95% CI [0.69, 0.83]) had the highest discriminatory power, followed by PCT (AUC = 0.65, 95% CI [0.57, 0.73]), LBP (AUC = 0.65, 95% CI [0.56, 0.73]), and SERPINA1 (AUC = 0.65, 95% CI [0.56, 0.73]) while SERPINA3 (AUC = 0.56, 95% CI [0.48, 0.65]) and HRG (AUC = 0.57, 95% CI [0.49, 0.65]) could not discriminate beyond chance (Supplementary Table S5). There was a high degree of overlap observed across the bacterial and viral biomarker levels (Figure 2). In assessing interactions between pneumonia severity and biomarkers, there was a significant interaction between pneumonia severity and AGT (RR = 0.85, *p*-value = 0.0080), CRP (RR = 0.77, *p*-value = 0.0073), HRG (RR = 0.85, *p*-value = 0.0380), and SERPINA1 (RR = 0.80, *p*-value = 0.0058) (Supplementary Table S8).

**Table 1:** Biomarker thresholds derived when comparing all possible bacterial cases and probable viral cases, and their sensitivity and specificity in differentiating between all possible bacterial cases and probable viral cases.

Biomarker	Threshold (MFI)	Sensitivity	Specificity	PPV	NPV	AUC [95% CI]
AGT	7.9144	0.49	0.72	0.64	0.58	0.60 [0.53, 0.66]
CRP	9.1514	0.90	0.19	0.53	0.66	0.54 [0.48, 0.61]
PCT	6.1021	0.24	0.90	0.71	0.82	0.58 [0.52, 0.64]
LBP	7.6617	0.33	0.84	0.68	0.78	0.56 [0.50, 0.63]
HRG	7.7132	0.64	0.46	0.54	0.62	0.53 [0.46, 0.59]
PON1	5.9797	0.66	0.45	0.55	0.62	0.54 [0.48, 0.61]
SERPINA1	8.9741	0.29	0.91	0.75	0.86	0.58 [0.52, 0.64]
SERPINA3	8.7465	0.39	0.70	0.57	0.66	0.53 [0.47, 0.59]

Area Under the Curve (AUC) represents the discriminatory power of each predictor in distinguishing bacterial from viral pneumonia. Sensitivity is the proportion of true bacterial cases accurately identified by each predictor. Specificity is the proportion of true viral cases accurately identified by each predictor. Positive Predictive Value (PPV) is the proportion of cases predicted as bacterial that are truly bacterial. Negative Predictive Value (NPV) is the proportion of cases predicted as viral that are truly not bacterial.

**Figure 2:**
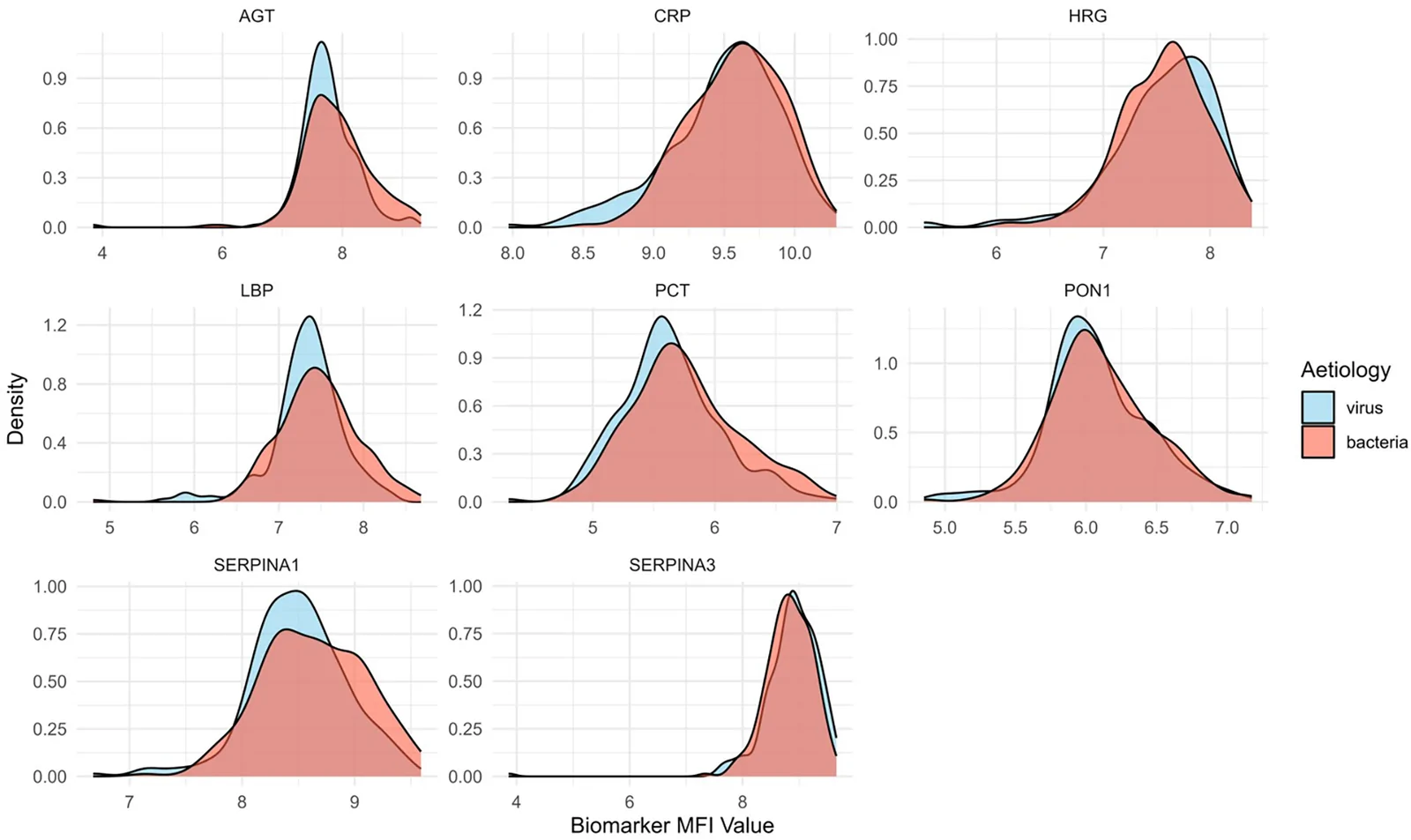
This is a density plot showing how the biomarker median fluorescent intensity (MFI) levels between the bacterial and viral groups of infection overlap.

### Modified poisson regression analyses

In age-adjusted analyses, restricted to the training set, mid-upper arm circumference, chest-wall indrawing, cough, convulsion, crackles, AGT (in continuous and categorical forms), and SERPINA1 (in a categorical form that used different cut-offs for children <1 and ≥1 year of age) were predictive of pneumonia etiology (Table 2). In multivariable analyses controlling for age, only lower chest-wall indrawing (LCWI) (aRR = 0.75, 95% CI [0.61, 0.93], *p*-value = 0.0081) remained a significant predictor, with a higher prevalence in the viral pneumonia group. After controlling for age and LCWI, none of the other clinical signs or symptoms, or biomarker levels, was predictive of etiology (Table 2).

**Table 2:** Predictors of pneumonia etiology identified in the training dataset using a multivariable poisson regression model.

Characteristic	Overall *N* = 319^a^	Probable viral *N* = 159^a^	Possible bacterial *N* = 160^a^	Crude RR [95% CI]	LRT *p*-value	Adjusted RR [95% CI]	LRT *p*-value
Age groups					0.7391		0.6998
2–5	98 (31%)	60 (38%)	38 (24%)	Ref		Ref	
6–11	86 (27%)	44 (28%)	42 (26%)	1.07 [0.84, 1.37]		1.04 [0.81, 1.33]	
12–23	72 (23%)	33 (21%)	39 (24%)	1.11 [0.86, 1.43]		1.08 [0.83, 1.40]	
24–35	35 (11%)	14 (8.8%)	21 (13%)	1.15 [0.84, 1.56]		1.18 [0.85, 1.62]	
>= 36	28 (8.8%)	8 (5.0%)	20 (13%)	1.24 [0.88, 1.70]		1.25 [0.88, 1.76]	
Chest-wall indrawing					0.0081		0.0081
No	65 (20%)	7 (4.4%)	58 (36%)	Ref		Ref	
Yes	254 (80%)	152 (96%)	102 (64%)	0.75 [0.61, 0.93]		0.75 [0.61, 0.93]	
Mid-upper arm circumference	13.00 (12.00, 14.20)	13.40 (12.00, 14.30)	13.00 (11.40, 14.00)	0.96 [0.92, 1.01]	0.0842		
Cough					0.0143		
No	54 (17%)	5 (3.1%)	49 (31%)	Ref			
Yes	265 (83%)	154 (97%)	111 (69%)	0.75 [0.60, 0.94]			
Convulsion					0.1456		
No	285 (89%)	153 (96%)	132 (83%)	Ref			
Yes	34 (11%)	6 (3.8%)	28 (18%)	1.23 [0.93, 1.59]			
Crackles					0.1218		
No	202 (63%)	83 (52%)	119 (74%)	Ref			
Yes	117 (37%)	76 (48%)	41 (26%)	0.86 [0.71, 1.04]			
AGT^1^	7.79 (7.52, 8.15)	7.70 (7.50, 8.04)	7.91 (7.52, 8.30)	1.12 [0.95, 1.32]	0.1786		
AGT categorical^b^					0.1119		
Below (<7.9144)	195 (61%)	114 (72%)	81 (51%)	Ref			
Above (>=7.9144)	124 (39%)	45 (28%)	79 (49%)	1.16 [1.08, 1.24]			
AGT (at one year cut-off)^c^					0.1077		
Low	182 (57%)	107 (67%)	75 (47%)	Ref			
High	137 (43%)	52 (33%)	85 (53%)	1.16 [0.97, 1.39]			
SERPINA1 (at one year cut-off)^d^					0.1053		
Low	227 (71%)	129 (81%)	98 (61%)	Ref			
High	92 (29%)	30 (19%)	62 (39%)	1.20 [0.94, 1.39]			
AGT (at two years cut-off)^e^					0.1514		
Low	166 (52%)	97 (61%)	69 (43%)	Ref			
High	153 (48%)	62 (39%)	91 (57%)	1.14 [0.95, 1.37]			
AGT (at three years cut-off)^f^					0.1581		
Low	164 (51%)	96 (60%)	68 (43%)	Ref			
High	155 (49%)	63 (40%)	92 (58%)	1.14 [0.95, 1.36]			

Variables significantly associated in crude analyses controlling for age (LRT *p*-value <0.2) were assessed for inclusion in the final multivariable model using backwards regression model building techniques. All crude RRs were adjusted for age.

a AGT MFI values in continuous form.

b AGT categorized into a binary variable based on the optimal threshold of the entire population under study.

c AGT categorized based on different optimal thresholds for <1 and ≥1 year of age.

d SERPINA1 categorized into a binary variable using different optimal thresholds for children <1 and ≥1 year of age.

e AGT categorized into a binary variable using different optimal thresholds for children <2 and ≥2 years of age.

f AGT categorized into a binary variable using different optimal thresholds for children <3 and ≥3 years of age. The full list of variables assessed in crude analyses are included in supplementary Table 4.

The multivariable model including age and LCWI exhibited moderate discrimination (AUC = 0.72, 95% CI [0.66, 0.77]), with a sensitivity of 0.55 and a specificity of 0.84 on the training dataset. The model had good calibration with a calibration intercept of 9.31e-12 (*p*-value = 1.0000) and a calibration slope of 1.000 (*p*-value = 0.0000). The model performed poorly on the validation dataset (AUC = 0.61, 95% CI [0.52, 0.70]; sensitivity 0.50, specificity 0.68) (Figure 3). The model exhibited some miscalibration in the validation dataset with a calibration intercept of −0.2379 (*p*-value = 0.1538) and a calibration slope of 0.5688 (*p*-value = 0.0074). Cross-validation was also performed to ascertain the stability of the model-building process. The five-fold AUCs ranged from 0.54 to 0.68 (Supplementary Table S9) with a mean validation AUC of 0.64 [0.56, 0.71]. Chest wall indrawing was retained in 3 out of 5 folds, cough and SERPINA1 (**>=**8.980252 MFI) were retained in 2 out of 5 folds, crackles and LBP (**>=**7.664855**)** were retained in 1 fold, while no variable was retained in 4 or more folds.

**Figure 3:**
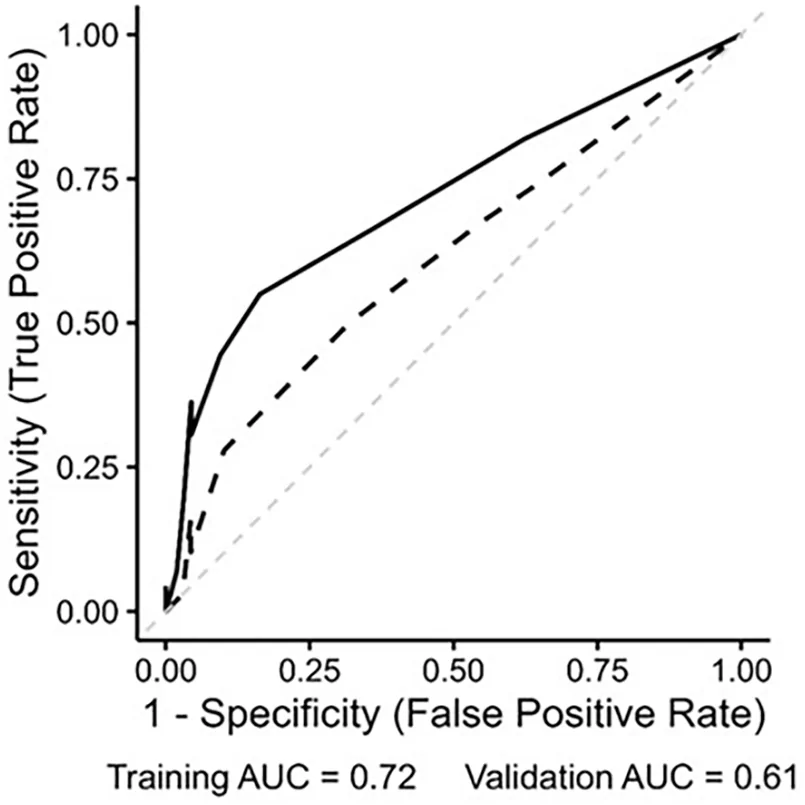
This is the receiver operating characteristic (ROC) curve showing the discrimination between the training and testing sets. This is the ability of the modified Poisson regression model to differentiate between the possible bacterial and viral cases of pneumonia.

In a separate analysis restricted to the training set, excluding unknown infections and adjusting for age, chest-wall indrawing [aRR = 0.24 (0.14, 0.41), *p*-value <0.0001], cough [aRR = 0.39 (0.27, 0.55), *p*-value = 0.0004], AGT (>=7.9144) [aRR = 2.92 (1.97, 4.30), *p*-value =  < 0.0001], and crackles [aRR = 0.21 (0.10, 0.43), *p*-value <0.0001] were significant predictors of pneumonia etiology in the multivariable model. The model showed very good discrimination in the training set, with an AUC of 0.91 [0.86, 0.96], a sensitivity of 0.77, and a specificity of 0.96. It was well calibrated, with a calibration intercept of 0 (*p*-value = 1.0000) and a calibration slope of 1.0 (*p*-value =  < 0.0001). When tested on the validation dataset, the model had moderate discrimination, with an AUC of 0.78 [0.67, 0.89], a sensitivity of 0.61, and a specificity of 0.96. However, the model was miscalibrated, with a calibration intercept of 0.0696 (*p*-value = 0.6235) and a calibration slope of 0.6915 (*p*-value = <0.0001).

### Comparison with other available models

A study conducted in Indonesia (15) by Farida et al. identified CRP (≥5.70 mg/L), presence of fever (≥38 °C), and rainy season as predictors of pneumonia aetiology. The combination of these three factors exhibited poor predictive capacity and could not discriminate beyond random chance in our validation study (AUC of 0.59 (95% CI [0.46, 0.71])). Bhuiyan et al.'s study (9) identified CRP (≥72 mg/L), fever (≥38 °C), and the presence or absence of rhinorrhea as significant predictors of aetiology. When these factors were combined, the model exhibited moderate predictive capacity in our validation dataset (AUC = 0.74, 95% CI [0.64, 0.85] with a sensitivity of 0.55 and a specificity of 0.86) (Supplementary Table S6).

## Discussion

In this nested case-control study, we utilized a dataset containing data on a wide range of clinical signs and symptoms, complete blood cell counts, CRP, PCT and 6 novel biomarker levels to develop a multivariable predictive model and evaluate its ability to discriminate between bacterial and viral pneumonia in children presenting with WHO-defined pneumonia. The model incorporated some novel biomarkers, which have not been assessed for this purpose before, to evaluate their incremental value in predicting etiology and to account for any underlying biological pathways not captured by conventional biomarkers. The majority of study participants were severely ill, reflecting the severe case mix presenting to the hospital in Kilifi, and more broadly, sub-Saharan Africa (24, 25), where morbidity is underpinned by poverty, malnutrition, and inaccessibility to healthcare facilities.

In age-adjusted analyses, higher MFI units of AGT, LBP, and SERPINA1 were associated with increased probability of an individual having bacterial pneumonia. However, neither biomarker remained significantly predictive of bacterial infections after controlling for age and chest-wall indrawing. In internal validation, the final model, including age and LCWI, lacked sufficient predictive power to guide clinical decision-making (AUC = 0.61, 95% CI [0.52, 0.70], sensitivity = 0.50, specificity = 0.68). Performing internal validation indicated that the predictions were overly extreme (potential model overfit), whereas the training set was well calibrated. This is consistent with the internal cross-validation model performance, which had a mean AUC of 0.64, indicating that model development performance was optimistic (AUC = 0.72), with overestimation on unseen observations. When we performed sensitivity analyses excluding participants with unknown etiology, model performance improved. This model, which included chest-wall indrawing, cough, crackles, and AGT (>=7.9144) and was adjusted for age, achieved an AUC of 0.91. These findings suggest that including participants with unknown infections may have attenuated the model's ability to distinguish bacterial from viral pneumonia.

Other published models developed for the same purpose have reported equally poor performance on internal validation. The performance of these models did not improve when we externally validated them on our dataset. The study by Farida et al. (15) was conducted in a population with a similar age group and case mix to our population. However, their outcome of interest was defined differently from ours as they excluded unknown infections. Despite this, their model performed just as badly on our population as in their own internal validation. The model identified by Bhuiyan et al. (9), despite having moderate discrimination in our study population (AUC = 0.74), lacks sufficient sensitivity to guide clinical decision-making (sensitivity = 0.55).

Differentiating bacterial from viral pneumonia remains a challenge. Incorporating novel biomarker levels in our study did not improve predictive performance compared with other models that included only clinical and demographic data. The distribution of MFI units significantly overlapped in bacterial and viral cases. To explore possible reasons why, after controlling for age and LCWI, biomarker levels were not predictive, we assessed interactions between biomarkers’ predictive capacity and pneumonia severity, adjusting forage. CRP, AGT, HRG, and SERPINA1 had significant interactions with pneumonia severity; higher values were more predictive of pneumonia etiology in non-severe cases than in severe cases (supplementary Table S8), as also reported by Wu et al. (26), who noted that CRP was significantly associated with severe pneumonia and therefore suboptimal as a stand-alone predictor. Other studies have found that levels of SERPINA1, PON1, and LBP are higher in bacterial infections and can identify patients with CAP among other patients (7, 19, 27–30), however, these markers do not adequately differentiate between bacterial and viral etiologies. HRG has been shown to exert an antifungal effect, and its levels negatively correlate with disease severity (31). Previous studies have reported interactions between CRP and age and LBP and age (16, 32–34). We assessed each biomarker using different cut-offs for different ages; however, this did not significantly improve their predictive capacity.

This analysis, as with others before it, has notable strengths and limitations. The strengths include: a representative sample of 457 pediatric hospital admissions, with biological samples which were tested for a wide range of bacterial and viral pathogens known to cause pneumonia at a world-class research laboratory. The available data on possible predictors included a wide panel of novel and conventional biomarkers (eight, 8) along with over 20 clinical signs and symptoms (25). Despite these strengths, we acknowledge that a major limitation is probable outcome misclassification: 54% of the children included in the possible bacterial group had an unknown etiology, with a negative blood culture and negative PCR for all tested viral targets. This is a direct result of the insensitivity of bacterial cultures and the specificity required by PCR (6, 35). Although bacterial culture is the gold standard for identifying bacterial infections, it is insensitive due to contamination, prior antibiotic therapy, and infections existing in inaccessible sites (32, 35). Furthermore, the PCR testing panel that was used to define viral pneumonia identified 11 respiratory pathogens. However, there are other viruses known to cause pneumonia-related hospitalizations, such as human bocavirus, which were not on the PCR testing panel. The viral infection outcome was identified from NP/OP swabs, which is only a proxy for the site of infection in the lungs, potentially leading to outcome misclassification.

An additional limitation of this analysis is that the laboratory biomarker levels were recorded as MFI units. Although internally valid, this limits the study's replicability, generalizability, and direct comparability to other studies that report their levels and biomarker thresholds as concentrations. Lastly, although our study cohort is representative of pediatric hospital admissions, a large amount of antimicrobial use occurs in outpatient settings, and our findings cannot be generalized to outpatient care.

### Conclusion

Distinguishing between bacterial and viral cases of childhood pneumonia in sub-Saharan Africa remains a challenge. This analysis follows the widespread administration of the PCV and Hib vaccines in Kenya; in this context, the case mix presentating to the hospital is increasingly of viral etiology and therefore would not benefit from antibiotics. However, in our analysis, which included all children presenting with WHO-defined pneumonia or severe pneumonia, including infections of unknown etiology, we still could not identify a group of predictors that can accurately identify bacterial infections with enough sensitivity and specificity to guide clinical decision-making. Our study has demonstrated that biomarker performance is affected by disease severity and thresholds could differ with age. Further research addressing some of the limitations we encountered in outcome classification, stratifying by disease severity and potentially accounting for variation in biomarker performance by age, may improve predictions.

## Data Availability

The data analyzed in this study is subject to the following licenses/restrictions: The data analysed in this study are not publicly available as they are owned by the KEMRI-Wellcome Trust Research Programme. Access is governed by the KEMRI-Wellcome Trust Research Programme Data Governance Committee. Researchers may request access from the author or the data governance office, subject to review and approval by the relevant data governance committee. Requests to access these datasets should be directed to dgc@kemri-wellcome.org.
